# Preventive medication efficacy after 1-year follow-up for graft failure in coronary artery bypass surgery patients: Bayesian network meta-analysis

**DOI:** 10.1093/ehjopen/oeae052

**Published:** 2024-06-27

**Authors:** Mikko Uimonen, Rasmus Liukkonen, Ville Ponkilainen, Matias Vaajala, Jeremias Tarkiainen, Oskari Pakarinen, Marjut Haapanen, Ilari Kuitunen

**Affiliations:** Tampere University Hospital, Heart Hospital, Elämänaukio 1, 33520 Tampere, Finland; Faculty of Medicine and Health Technology, Tampere University, Arvo Ylpön katu 34, 33520 Tampere, Finland; Faculty of Medicine and Health Technology, Tampere University, Arvo Ylpön katu 34, 33520 Tampere, Finland; Department of Surgery, Central Finland Hospital Nova, Jyväskylä, Finland; Faculty of Medicine and Health Technology, Tampere University, Arvo Ylpön katu 34, 33520 Tampere, Finland; Faculty of Medicine and Health Technology, Tampere University, Arvo Ylpön katu 34, 33520 Tampere, Finland; Department of Surgery, Päijät-Häme Central Hospital, Lahti, Finland; Institute of Clinical Medicine, University of Eastern Finland, Kuopio, Finland; Institute of Clinical Medicine, University of Eastern Finland, Kuopio, Finland; Department of Pediatrics, Kuopio University Hospital, Kuopio, Finland

**Keywords:** Graft failure, Coronary artery bypass grafting, Antiplatelet, Dual antiplatelet therapy

## Abstract

To compare preventive medications against graft failures in coronary artery bypass graft surgery (CABG) patients after a 1-year follow-up. Systematic review with Bayesian network meta-analysis and meta-regression analysis. We searched PubMed, Scopus, and Web of Science databases in February 2023 for randomized controlled trials, comparing preventive medications against graft failure in CABG patients. We included studies that reported outcomes at 1 year after surgery. Our primary outcome was graft failure After screening 11,898 studies, a total of 18 randomized trials were included. Acetylsalicylic acid (ASA) [odds ratios (OR) 0.51, 95% credibility interval (CrI) 0.28–0.95, meta-regression OR 0.54, 95% CrI 0.26–1.00], Clopidogrel + ASA (OR 0.27, 95% CrI 0.09–0.76, meta-regression OR 0.28, 95% CrI 0.09–0.85), dipyridamole + ASA (OR 0.50, 95% CrI 0.30–0.83, meta-regression OR 0.49, 95% CrI 0.26–0.90), ticagrelor (OR 0.40, 95% CrI 0.16–1.00, meta-regression OR 0.43, 95% CrI 0.15–1.2), and ticagrelor + ASA (OR 0.26, 95% CrI 0.10–0.62, meta-regression OR 0.28, 95% CrI 0.10–0.68) were superior to placebo in preventing graft failure. Rank probabilities suggested the highest likelihood to be the most efficacious for ticagrelor + ASA [surface under the cumulative ranking (SUCRA) 0.859] and clopidogrel + ASA (SUCRA 0.819). The 95% CrIs of ORs for mortality, bleeding, and major adverse cardio- and cerebrovascular events (MACE) were wide. A trend towards increased bleeding risk and decreased MACE risk was observed when any of the medication regimens were used when compared to placebo. Sensitivity analysis excluding studies with a high risk of bias yielded equivalent results. Of the reviewed medication regimens, dual antiplatelet therapy combining ASA with ticagrelor or clopidogrel was found to result in the lowest rate of graft failures.

## Introduction

Despite the increasing number of percutaneous coronary interventions performed worldwide, coronary artery bypass grafting (CABG) still remains a recommended treatment for patients with moderate to complex coronary artery disease.^1,2^ The bypassing may be performed using grafts such as internal thoracic arteries, radial artery, gastroepiploic artery or saphenous vein. Along with anastomosing the left internal thoracic artery to the left anterior descending coronary artery, saphenous vein is still the most common graft for secondary anastomoses.^3–7^ However, anastomosing saphenous vein to coronary arteries encompasses an increased risk for graft stenosis and failure when compared to arterial grafts, and up to 30–40% of saphenous vein grafts (SVG) have been reported to fail within a year from surgery.^8,9^ To improve saphenous graft patency, the use of antiplatelet medication, most commonly acetylsalicylic acid (ASA), is an established practice after CABG.^1,2,10,11^ However, as a cost of improved SVG patency, these medications possess an increased risk for bleeding complications.^12–14^

There are several agents available to improve SVG patency, and the use of double agents has gained increasing attention. During the previous decades, the body of knowledge has been increasing along with several randomized controlled trials, meta-analyses and, during the past few years, network meta-analyses.^15–17^ Still, uncertainty remains on which medication regimen would be the most beneficial, and the previous evidence-composing reviews have raised some methodological concerns affecting the applicability of the results.

First, there has been a high variability in follow-up times in studies in which results have been pooled in the previous network meta-analyses.^15–17^ In these works, the minimum follow-up period has either not been defined or has been limited to more than 3 months. However, with regards to the pathophysiology of SVG failure, early failures are predominantly related to operative and technical factors of CABG surgery and competitive flow rather than sporadic thrombosis, intimal hyperplasia and atherosclerosis, of which risk may be modified by appropriate preventive medication.^3^ Further, due to the slow pace of the pathophysiological process leading to SVG occlusion, the consequences of disease progression become visible after several months.^3^ Therefore, the efficacy of preventive should be assessed no earlier than 1 year from surgery and, subsequently, it is clear, that 3-month follow-up is too short.

Second, there have been discrepancies in the outcome measure definitions. The SVG failure rate may be reported per patient, per graft or per distal anastomosis. The previous network meta-analyses have either inappropriately pooled per patient and per graft data leading to guaranteed inaccuracy in the effect estimates or studied outcomes per graft only without accounting per patient data.^15–17^

Third, since the early course of SVG failure prevention trials several decades ago, there have been massive advances in operative techniques, equipment, perioperative care, and guidelines. Additionally, there may be differences in patient-related and institutional factors related to SVG failure rates between trials. These factors may have influenced the effect estimates in individual studies due to which it would be beneficial to conduct a meta-regression analysis with appropriate covariates.

With an appreciation of these aspects of previous literature, we conducted a Bayesian network meta-analysis and meta-regression to compare different medication regimens to prevent SVG failures that are not related to technical factors or competitive flow with a follow-up of 1 year.

## Methods

### Search and screening process

Search for this systematic review was performed on 19 February 2023. PubMed, Scopus, and Web of Science databases were searched from inception. The search strategy is described in Supplementary material online, *Appendix S1*. All authors contributed to the abstract screening and each abstract was screened by two individual authors. Abstracts accepted by both authors were included in full-text screening. Cases of discrepancy between the screening authors were solved by third author. Full report screening was carried out in a similar manner. Covidence software was used in the screening process. We did not search grey literature. The reference lists of the included studies were screened manually to find missed relevant studies for inclusion. Previous meta-analyses were also searched for relevant studies.

### Inclusion and exclusion criteria

We included randomized controlled trials comparing medical treatment regimens to prevent vein graft failure after CABG surgery and reporting outcomes at 12 months from the surgery. All studies that reported observational data or did not report original data were excluded.

### Patients

Patients were required to undergo CABG surgery due to coronary artery disease. Both chronic and acute-phase surgery patients were included. CABG surgery was defined as cardiac surgery procedure in which the blood flow of an occluded coronary artery has been restored by bypassing the occluded segment of the artery using a vein graft. Percutaneous coronary interventions were excluded.

### Intervention

We included all medical treatment regimens that are targeted against vein graft failure including agents affecting blood coagulation and thrombus formation. The minimum number of patients per treatment regimen in all included studies was set to 100 and those regimens with less than 100 patients were excluded.

### Comparator

Placebo was used as a comparator for all studied medication regimens in our network analysis.

### Outcomes

Our main outcome was the graft failure which was defined as graft occlusion or thrombosis. Stenosis of anastomosis without occlusion was not considered as graft failure. The follow-up time was set to 12 months. Secondary outcomes were mortality, bleeding complications requiring intervention and major adverse cardio- or cerebrovascular events (MACE) during the 12 months of follow-up.

### Data extraction

The following information was extracted from each study: authors, funding, competing interests, inclusion and exclusion criteria, study period, country, intervention definition, control definition, outcome definitions, number of included patients, number of events, and main outcome measures.

### Evidence certainty

Evidence certainty was assessed by CINeMA (Confidence in Network Meta-Analysis) frameworks.^18,19^ Evidence certainty was ranked from very low to high.

Within-study bias was assessed by two authors independently according to Cochranes Risk of bias 2.0 tool.^20^ Risk of bias plots were generated using Robvis shinyapp.^21^ In the risk of bias assessment, the lack of blinding was not judged as an issue as the outcome assessment was considered not to be influenced by the knowledge of the intervention. Thus, we have utilized the same risk of bias assessment in all outcomes, as per the recommendation suggests considering the risk of bias for each outcome.

Reporting bias was considered generally low in all studies as no evidence of selective reporting or unpublished reports was noticed. Due to strict inclusion criteria with clearly defined outcome variables, we considered that indirectness was not an issue. Imprecision, heterogeneity and incoherence were assessed according to CINeMA framework.^19^ OR values 0.90–1.10 set as a range of equivalence.^19^

### Statistical methods

We conducted a Bayesian network meta-analysis with four Markov chains. Due to the assumed between-study heterogeneity, we selected the random-effects model as our approach. The posterior distributions were estimated using Monte Carlo simulations. Simulation was performed in two phases. First, 5000 burn-in simulation iterations were performed to adapt the algorithm after which the results of these iterations were discarded. Second, 100 000 inference simulation iterations were performed to estimate the posterior distributions. Convergence of the algorithm was assessed by inspecting trace plots and calculating potential scale reduction factor (PSRF) values. PSFR values below 1.05 were considered to represent sufficient convergence. Inconsistency was assessed by conducting a node-splitting analysis. Non-significant differences (*P* > 0.05) between the direct and indirect effect estimates were interpreted as representing sufficient consistency of the network model.

After compiling the model, the crude effect estimates for each medication regimen were calculated as odds ratios (OR) along with 95% credibility intervals (95% CrI) with placebo set as a control treatment. The rank probabilities indicating the probability for each treatment to be the most efficacious were calculated. Further, the surface under the cumulative ranking (SUCRA) scores were calculated. Higher SUCRA score indicate higher likelihood of the medication regimen to be the most efficacious. A meta-regression analysis was performed to adjust for the between-study differences. Covariates included the mean age of patients, the proportion of female patients, the proportion of patients with acute coronary syndrome at the time of surgery, the proportion of patients operated using cardiopulmonary bypass, mean number of grafts used, number of years from study publication and overall failure rate in each study. The ‘failures per patient’ and ‘failures per graft’ analyses were performed separately. Sensitivity analysis was performed excluding high risk of bias studies. Since placebo was not studied in the low and moderate risk of bias studies, ASA was set to a control treatment in the sensitivity analysis.

Statistical analysis was performed using R statistical software [version 4.3.1, R Core Team (2023), R Foundation for Statistical Computing, Vienna, Austria].

This study has been conducted according to the guidelines in Cochrane handbook and reported according to the Preferred reporting items in systematic reviews and meta-analysis (PRISMA) guideline.^22,23^

### Protocol registration

Protocol was registered to International prospective register of systematic reviews (PROSPERO; ID: CRD42023482354; available from: https://www.crd.york.ac.uk/prospero/display_record.php?ID=CRD42023482354).

## Results

### Search results

**Figure 1:**
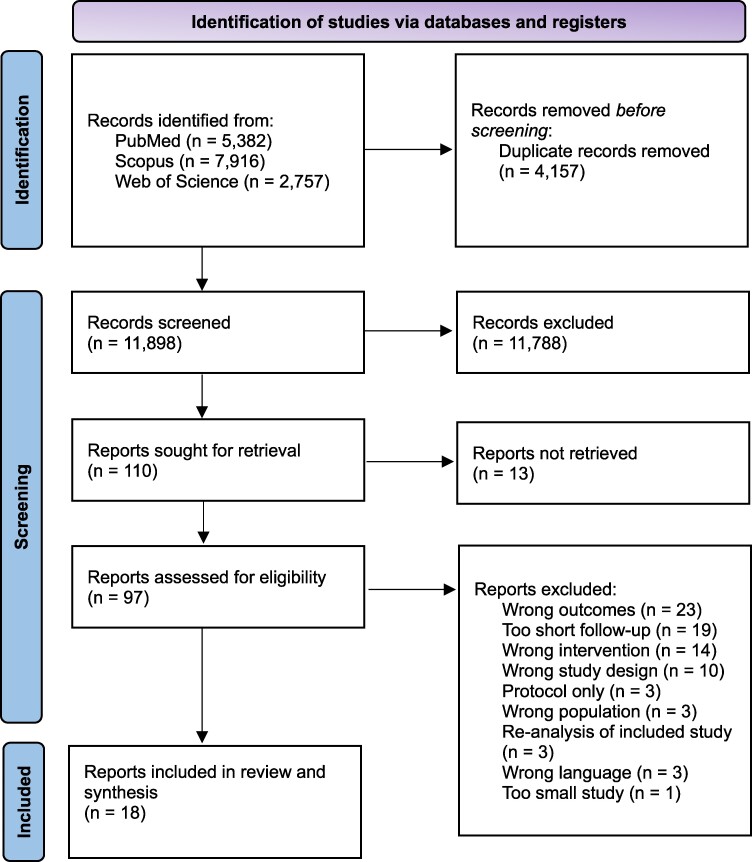
PRISMA flowchart of the study selection process.

The initial search provided a total of 11 898 studies after removing duplicates. After the screening, 18 studies were included in the synthesis (*Figure 1*).

### Study and patient characteristics

**Figure 2:**
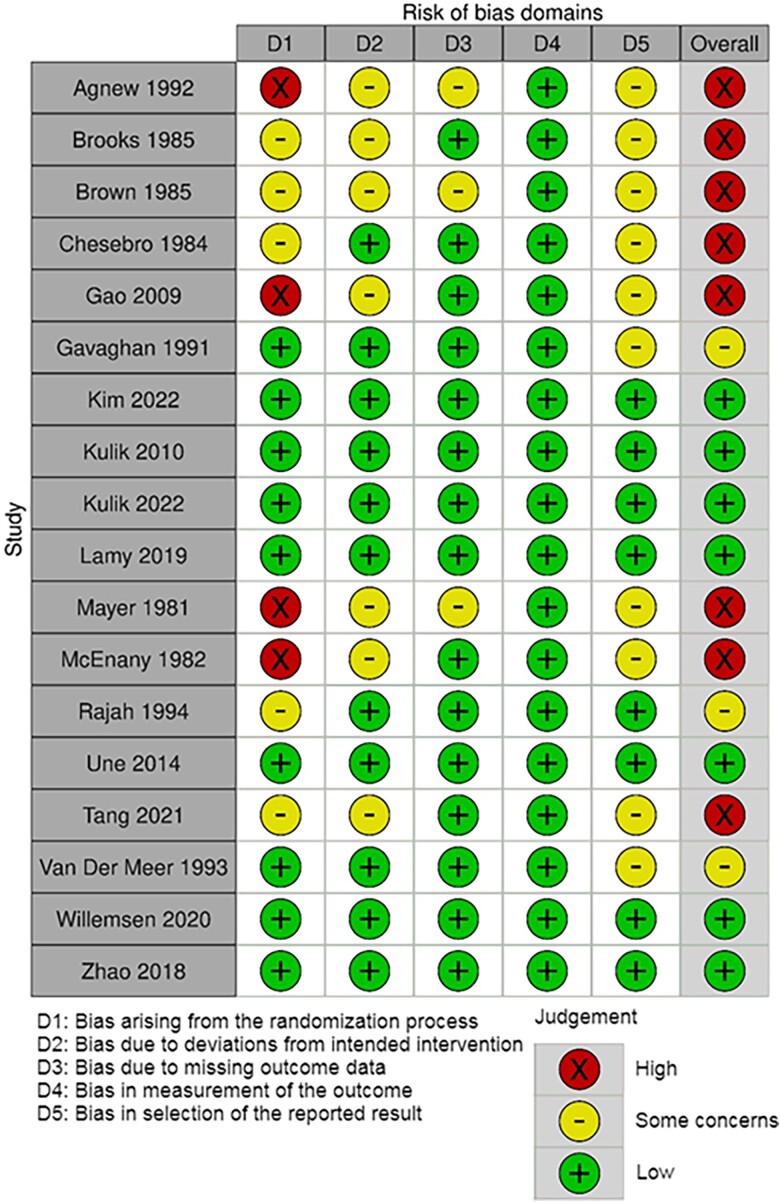
Within-study risk of bias of the included studies.

Study characteristics are presented in *Table 1*. The mean age of patients varied between 50 and 68 and the proportion of female varied between 0% and 39% across the studies. The mean number of bypassed coronary arteries varied from 1.9 to 3.8 between studies. Of the included studies, the overall risk of bias was low in seven, had some concerns in three, and was high in eight studies (*Figure 2*). Most issues were due to bias in the selection of the reported results and bias arising from the randomization process and from selection of the reported results. Confidence ratings according to CINeMA framework for the effects of each medication regimen in relation to placebo are presented in Supplementary material online, *Appendix S2* and *S3* for per patient and per graft analyses, respectively.

**Table 1:** Study characteristics

Study	Country	Study period	Funding	COI	Blinding	Follow-up (months)	Overall mean age	Overall women (%)	Patients with acute coronary syndrome at the time of surgery (%)	Patients operated using cardiopulmonary bypass(%)	Intervention	Mean number of bypassed coronary arteries per patient	Number of patients	Number of patients with graft failure (%)	Number of grafts	Number of failed grafts (%)
Agnew et al.^24^	New Zealand	1986–1988	Reported	Not reported	Double	12 months	56	5	0	100	Dipyridamole 300 mg × 1 + ASA 100 mg × 1	3.0	30		96	10 (10)
											ASA 100 mg × 1	3.0	31		96	10 (10)
Brooks et al.^25^	UK	1978–1982	Reported	Not reported	Double	12 months	54	12	0	100	Dipyridamole 75 mg × 3 + ASA 330 mg × 3	3.5	133	33 (25)	360	39 (11)
											Placebo	3.2	133	42 (32)	352	46 (13)
Brown et al.^26^	USA	1976–1980	Not reported	Not reported	Double	12 months	Not reported (range 34–70)	0	0	100	Dipyridamole 75 mg × 3 + ASA 325 mg × 3	3.1	45	15 (33)		
											ASA 325 mg × 3	3.1	38	10 (26)		
											Placebo	3.3	44	14 (32)		
Chesebro et al.^27^	USA	1977–1981	Reported	Not reported	Double	12 months	56	10	0	100	Dipyridamole 75 mg × 1 + ASA 325 mg × 1	2.8	171	37 (22)	478	53 (11)
											Placebo	2.8	172	81 (47)	486	121 (25)
Ekeström et al.^28^	Sweden	1983–1987	Reported	Not reported	Double	12 months	58	12	0	100	Dipyridamole 100 mg × 1	3.2	126		146	35 (24)
											Placebo	3.4	129		147	43 (29)
Gao et al.^29^	China	2005–2007	Not reported	Not reported	Single	12 months	62	17	0	63	Clopidrogel 75 mg × 1 + ASA 100 mg × 1	2.7	95	10 (11)		
											Clopidogrel 75 mg × 1	2.5	102	13 (13)		
Gavaghan et al.^30^	Australia	1984–1987	Not reported	Not reported	Double	12 months	56	26	0	100	ASA 324 mg × 1	3.4	119	7 (5.9)		
											Placebo	3.6	100	12 (12)		
Kim et al.^31^	Republic of Korea	2014–2020	Reported	None declared	Open-label	12 months	68	22	0	0	Ticagrelor 90 mg × 2 + ASA 100 mg × 1	3.1	102	4 (3.9)		
											Clopidogrel 75 mg × 1 + ASA 100 mg × 1	3.2	102	6 (5.9)		
Kulik et al.^32^	Canada	2006–2009	Reported	None declared	Double	12 months	67	11	19	96	Clopidogrel 75 mg × 1 + ASA 162 mg × 1	3.6	46	6 (13)		
											ASA 162 mg × 1	3.4	45	6 (13)		
Kulik et al.^33^	Canada	2014–2019	Reported	None declared	Double	12 months	68	16	61	88	Ticagrelor 90 mg × 2	2.9	100	17 (17)	289	30 (10)
											ASA 81 mg × 2	3.0	102	24 (24)	299	34 (11)
Lamy et al.^7^	Canada	2015–2017	Reported	None declared	Double	12 months	66	19	22	76	Rivaroxaban 2,5 mg × 2 + ASA 100 mg × 1	3.1	396	86 (22)	1242	113 (9.1)
											ASA 100 mg × 1	3.1	362	75 (21)	1154	92 (8.0)
											Rivaroxaban 2,5 mg × 2	3.1	381	68 (18)	1166	91 (7.8)
Mayer et al.^34^	USA	1973–1975	Not reported	Not reported	Double	12 months	54	18	0	100	Dipyridamole 50 mg × 2 + ASA 650 mg × 2	2.0	47	6 (13)	93	6 (6.5)
											Placebo	1.8	66	20 (30)	120	22 (18)
McEnany et al.^35^	USA	1979–1981	Reported	Not reported	Double	12 months	50	9	13	100	ASA 300 mg × 2	2.1	40	15 (37)	81	16 (20)
											Placebo	2.0	37	16 (43)	75	20 (27)
Mulder et al.^36^	Netherlands	1987–1990	Not reported	Not reported	Double	12 months	58	15	0	100	Dipyridamole 200 mg × 1 + ASA 50 mg × 1	3.6	30		107	24 (22)
											ASA 50 mg × 1	4.2	31		129	31 (24)
											Acenocoumarol or phenprocoumon (individual dosing)	5.0	31		154	41 (27)
Rajah et al.^37^	UK	1986–1989	Not reported	Not reported	Double	12 months	55	14	0	100	Indobufen 200 mg × 2	3.2	278	123 (44)	883	163 (18)
											Dipyradimole 75 mg × 3 + ASA 300 mg × 3	3.2	274	112 (41)	870	144 (17)
Tang et al.^38^	China	2017–2018	Reported	None declared	Open-label	12 months	64	39	0	69	Ticagrelol 90 mg × 2 + ASA 100 mg × 1	3.2	70		224	15 (6.7)
											Clopidogrel 75 mg × 1 + ASA 100mg	3.3	77		253	19 (8.7)
Une et al.^39^	Canada	2006–2009	Reported	None declared	Double	12 months	66	12	0	90	Clopidrogel 75 mg × 1 + ASA 162 mg × 1	Not reported	46	4 (8.7)		(4.8)
											ASA 162mg	Not reported	46	14 (30)		(4.5)
van der Meer et al.^40^	Netherlands/Switzerland/Germany	1987–1990	Reported	Not reported	Double	12 months	58	5	0	100	ASA 50 mg × 1	3.6	270	73 (27)	440	88 (20)
											Dipyridamole 200 mg × 2 + ASA 50 mg × 1	3.9	249	65 (26)	461	69 (15)
											Acenocoumarol 4 mg × 1 or phenprocoumon 6 mg × 1	3.8	257	69 (27)	448	85 (19)
Willemsen et al.^41^	Netherlands	2015–2019	Reported	Reported	Double	12 months	68	13	31	95	Ticagrelol 90 mg × 2 + ASA 80–100 mg × 1	3.7	219	26 (12)	457	44 (9.6)
											ASA 80–100 mg × 1	3.8	224	32 (14)	497	50 (10)
Zhao et al.^6^	China	2014–2015	Reported	Reported	Open-label	12 months	64	20	4	24	Ticagrelor 90 mg × 2 + ASA 100 mg × 1	3.8	168	30 (18)	458	29 (6.3)
											ASA 100 mg × 1	3.8	166	58 (35)	436	73 (17)
											Ticagrelor 90 mg × 2	3.8	166	49 (30)	445	55 (12)

### Model diagnostics

Trace plot and PSRF values indicated acceptable convergence of the model with the selected burn-in and inference simulation iterations as all PSRF values estimated at 1.000 (upper bound of PSRF 95% confidence interval range 1.00–1.01). The node-splitting analysis showed no prominent differences (*P* > 0.05) between the direct and indirect effect estimates for any of the medication regimens indicating sufficient consistency of the algorithm.

### Primary outcomes

#### Failures per patient

**Figure 3:**
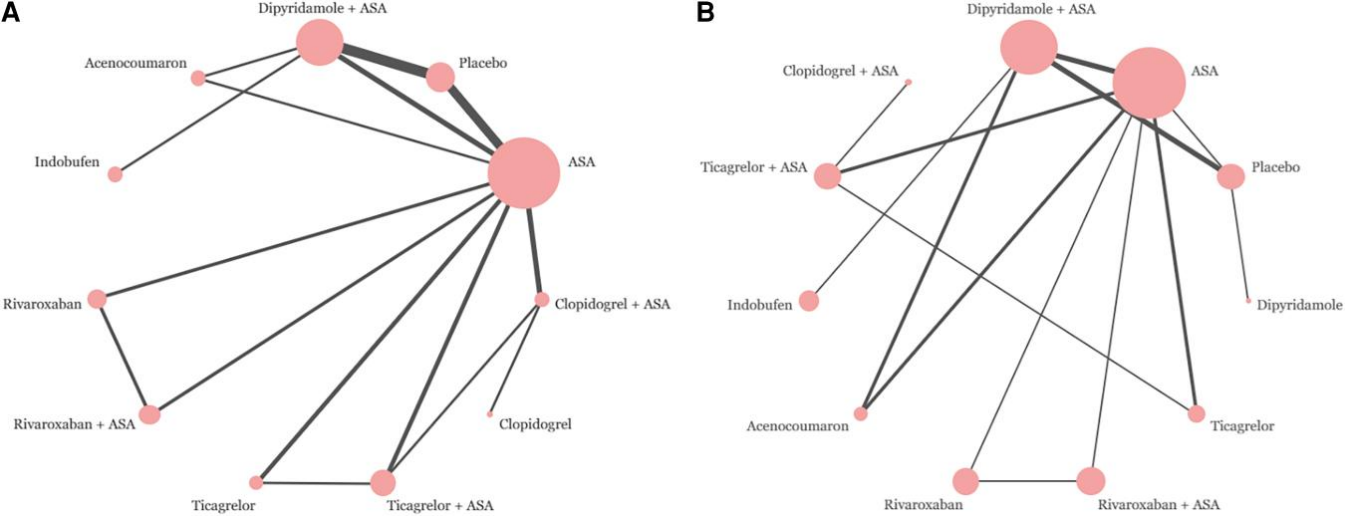
The network plots between the medication regimens. (*A*) The network of graft failures per patient. (*B*) The network of graft failures per graft. The edge width corresponds with the number of studies on the given comparison.

**Figure 4:**
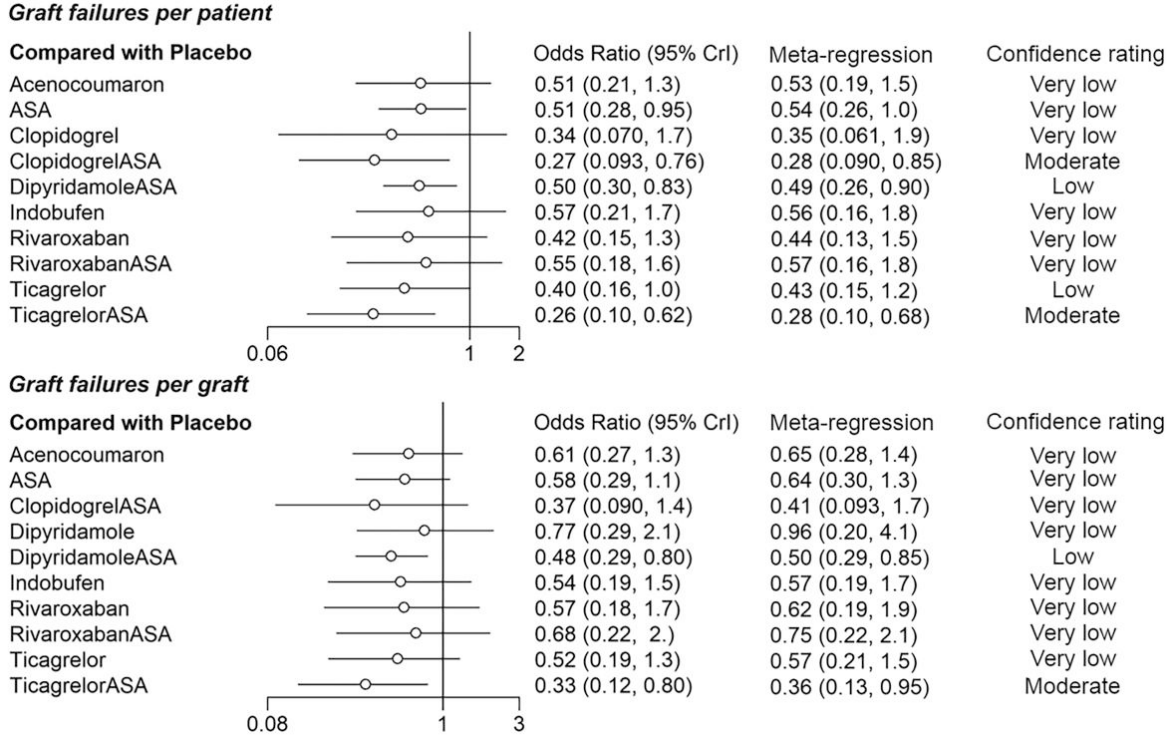
Crude and adjusted odds ratios for graft failure per patient and per graft of each medication regimen with placebo set as a control treatment. 95% Crl, 95% credibility interval.

**Figure 5:**
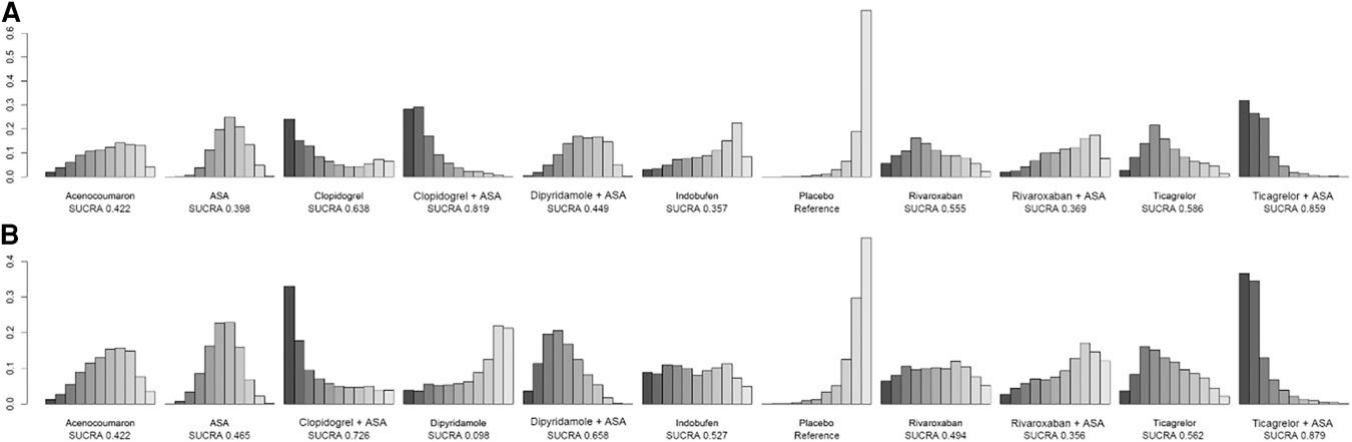
Rank probability distributions for each medication regimen. (*A*) Graft failures per patient. (*B*) Graft failures per graft. The leftmost bar signifies the first rank, i.e. the relative probability be the most efficacious medication regimen whereas the rightmost bar signifies the last rank, i.e. the relative probability to be the least efficacious regimen. The height of a bar shows the probability of the given rank. The surface under the cumulative ranking (SUCRA) score indicates the likelihood of a medication regimen to be the most efficacious with higher value indicating higher likelihood.

**Figure 6:**
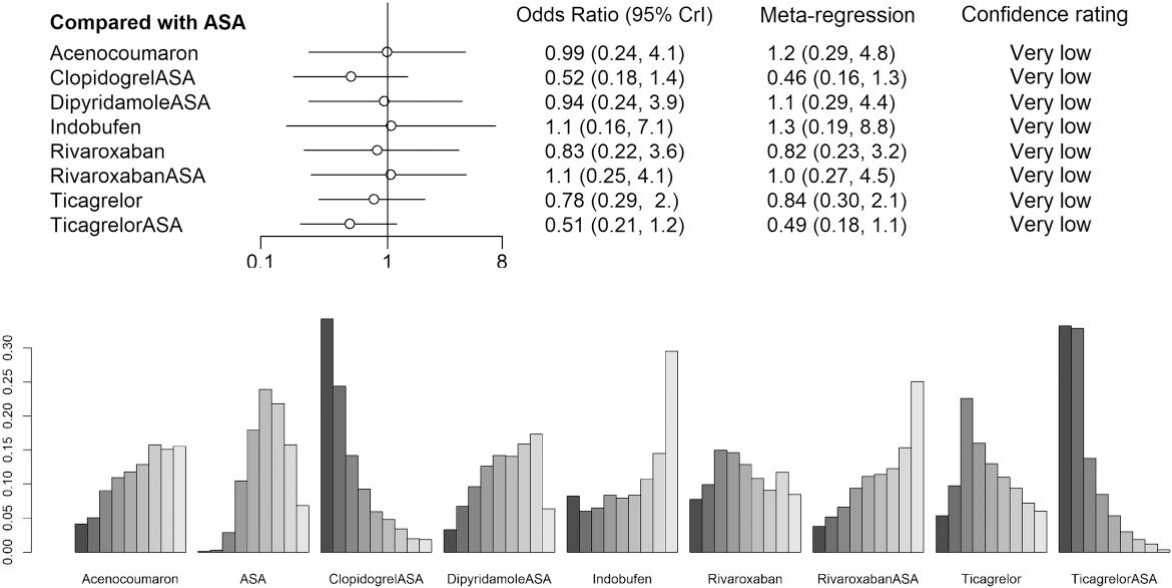
Sensitivity analysis of low risk of bias studies only for per-patient data.

The per-patient data involved overall 5341 patients from 16 studies. A total of 1278 (24%) patients had graft failure. Eleven medication regimens were included in the analysis (*Figure 3*). The ORs of ASA, clopidogrel + ASA, dipyridamole + ASA, ticagrelor and ticagrelor + ASA showed these medication regimens to be superior to placebo by the means of the 95% credibility level (*Figure 4*). Rank probabilities suggested the superiority of ticagrelor + ASA (SUCRA 0.859) and clopidogrel + ASA (SUCRA 0.819) over other medication regimens in preventing graft failures per patient (*Figure 5*). Of single agents clopidogrel (SUCRA 0.638), ticagrelor (SUCRA 0.586), and rivaroxaban (SUCRA 0.555) were found the most effective. The results of the meta-regression analysis showed similar findings. Confidence ratings of the medication regimens’ observed effects with placebo set as a reference were mainly low to very low while the effect of ticagrelor + ASA and clopidogrel + ASA rated as moderate confidence. Sensitivity analysis with high risk of bias studies excluded showed, according to the rank probabilities, tendency towards superiority of ticagrelor + ASA and clopidogrel + ASA over the other medications in line with the main analysis (*Figure 6*). However, the evidence on differences between the medication regimens was inadequate, given the wide CrIs of ORs.

#### Failures per graft

**Figure 7:**
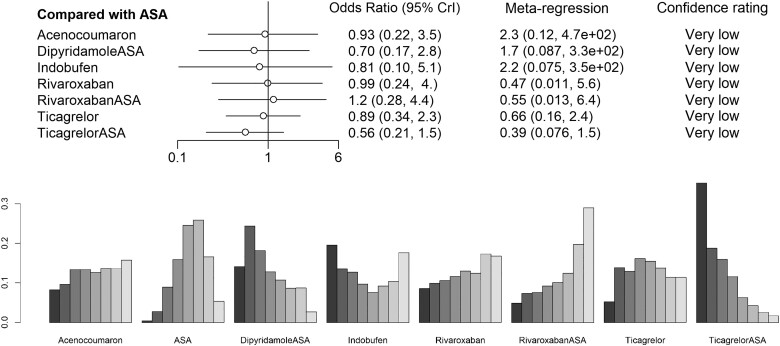
Sensitivity analysis of low risk of bias studies only for per-graft data.

Analysis of per-graft data involved a total of 12 942 grafts and 1711 observed graft failures in 14 studies. Ten medication regimens were analysed (*Figure 3*). The ORs of dipyridamole + ASA and ticagrelor + ASA appeared to be superior to placebo by means of the 95% credibility level (*Figure 4*). Rank probabilities suggested the superiority of ticagrelor + ASA (SUCRA 0.879) and clopidogrel + ASA (SUCRA 0.726) over the other medication regimens (*Figure 5*). Of single agents, ticagrelor (SUCRA 0.562) was found superior to other agents. The meta-regression analysis resulted in similar findings. Confidence ratings of the medication regimens’ observed effects with placebo set as a reference were generally low to very low while only the effect of ticagrelor + ASA rated as moderate confidence. Confidence ratings of the medication regimens’ observed effects with placebo set as a reference were generally very low except in ticagrelor + ASA and dipyridamole + ASA, which were rated as moderate and low confidence, respectively. In line with the main analysis, sensitivity analysis with a high risk of bias studies excluded showed, according to the rank probabilities, tendency towards superiority of ticagrelor + ASA over the other medications (*Figure 7*). In a light of wide CrIs of ORs, however, the evidence on differences between the medication regimens was insufficient.

### Secondary outcomes

**Figure 8:**
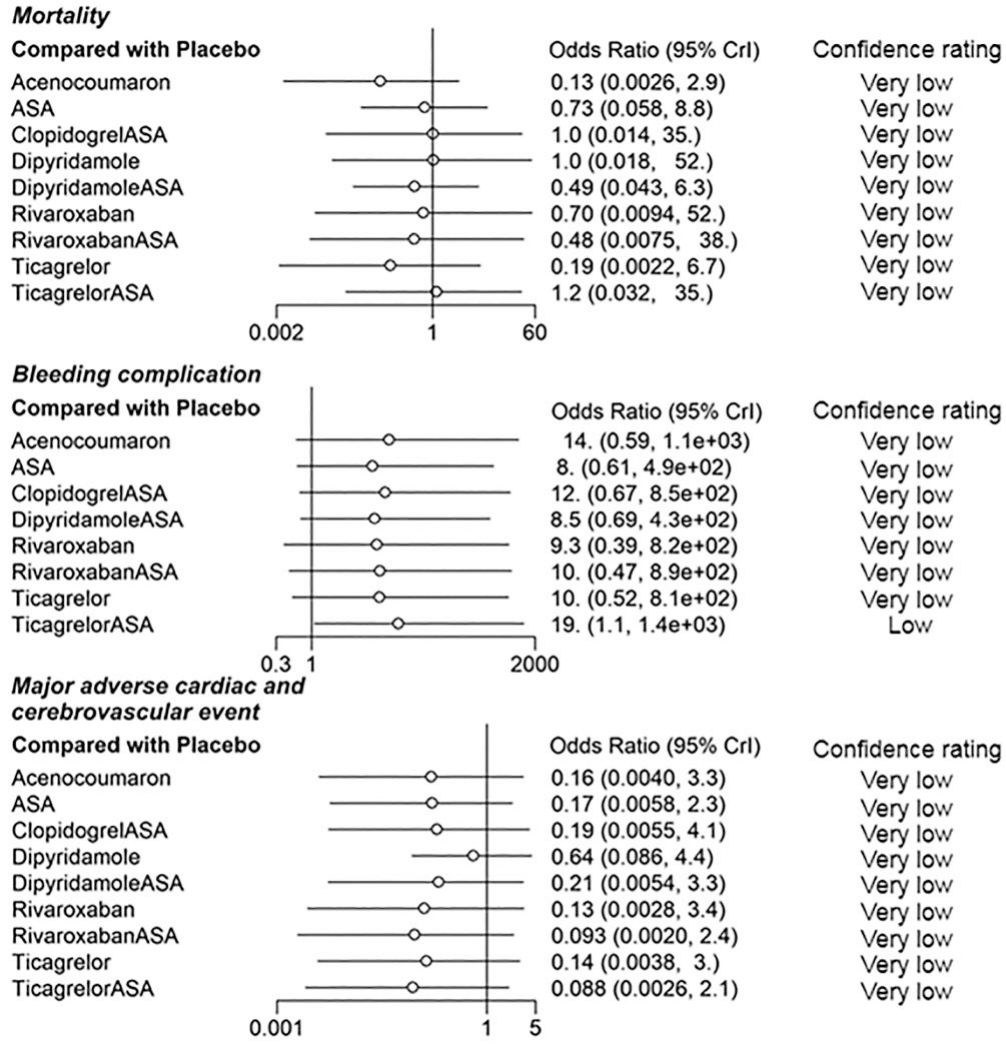
Odds ratios for complications of each medication regimen with placebo set as a control treatment. 95% Crl, 95% credibility interval.

**Figure 9:**
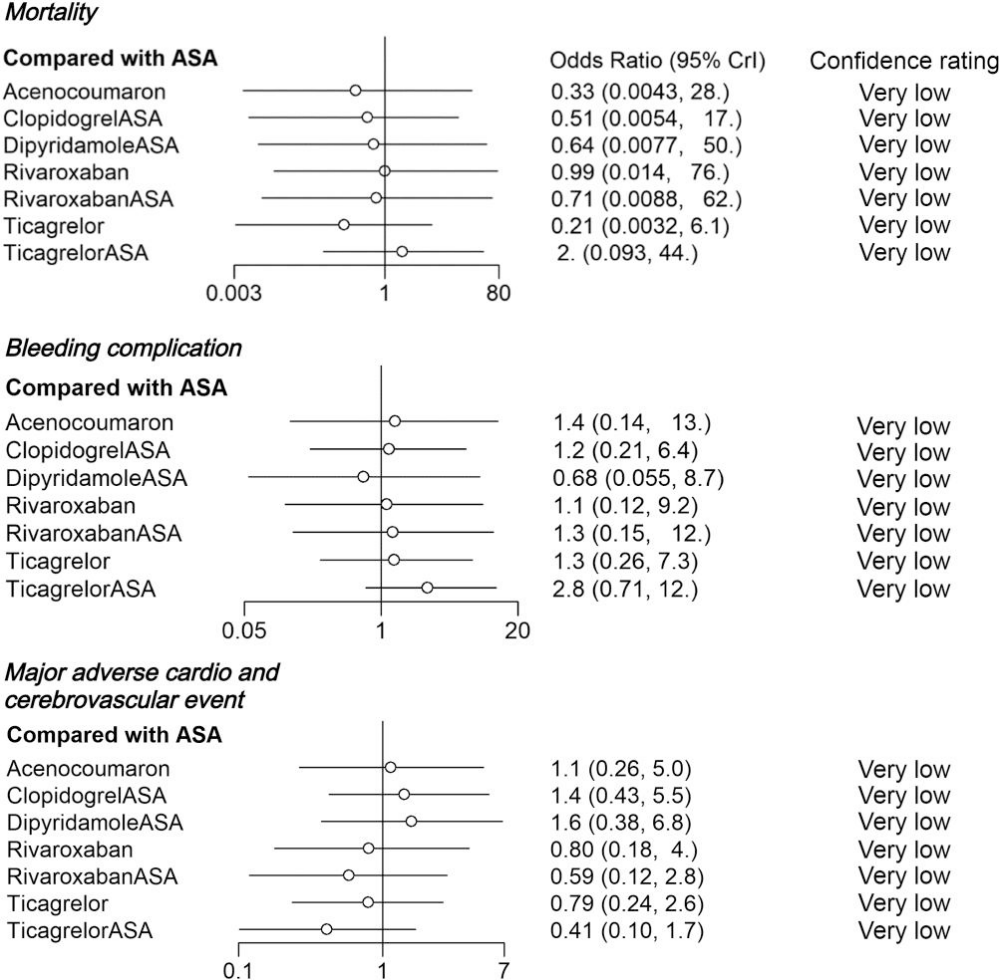
Sensitivity analysis of complications with high risk of bias studies excluded. ASA is set as a control treatment. 95% Crl, 95% credibility interval.

The 95% CrIs of ORs for mortality, bleeding and MACE were extremely wide (*Figure 8*). Although all the point estimates of ORs for bleeding complications showed increased risk and those for MACE showed decreased risk when compared to placebo, eligible evidence of increased complication risk was observed only in patients using ticagrelor + ASA (confidence rated as low), among which the OR indicated increased risk for bleeding complications. In the sensitivity analyses with high risk of bias studies excluded and ASA set as a reference, no eligible evidence of differences between the medication regimens was observed (*Figure 9*).

## Discussion

According to the results of this network meta-analysis and meta-regression, double antiplatelet treatment regimens ticagrelor + ASA and clopidogrel + ASA are most likely the most effective medication regimens to prevent SVG failure at 1 year from surgery, independent of whether the analysis was performed per patient or per graft. Of single agents, clopidogrel, ticagrelor, and rivaroxaban were found most likely the most effective although wide CrIs suggested high uncertainty of relative effectiveness between the studied agents. With regards to complications, uncertainty was high as observed by wide CrIs. However, evidence on increased bleeding risk related to ticagrelor + ASA combination was observed. These findings may be used in the assessment of risk for SVG failure and in planning of optimal medication regimen based on clinical and patient-related risk factors.

In the concurrent guidelines, ASA as a monotherapy is recommended as SVG failure preventing medication in CABG patients with stable coronary artery disease whereas in patients undergoing CABG due to acute coronary syndrome dual antiplatelet therapy should be used at least 1 year from surgery.^1,2,10^ However, evidence on benefits of dual antiplatelet therapy in CABG patients with stable coronary artery disease is scarce although there are some data suggesting that dual antiplatelet therapy may decrease SVG failure when compared to ASA monotherapy.^2,11^ With the existing knowledge gap, recommendations on antithrombotic medication after CABG in the current guidelines are cautious.^1,2,10^

While ASA monotherapy is a widely established practice recommended by the guidelines, there are some evidence suggesting benefit of dual antiplatelet therapy in certain patient groups. Beneficial effects seem more pronounced in patients undergoing off-pump CABG and when vein grafts are used.^42–44^ This in addition to the data suggesting higher graft failure rates in off-pump CABG especially when SVGs have been used, advocates towards favouring of dual treatment in these patients.^42–44^ With regards to the lacking guidelines, these findings underline the need of risk-benefit-assessment accounting individual patient characteristics when planning the medication strategy against graft failure after CABG. Moreover, some authors have even raised a concern on underutilization of dual treatment in coronary artery disease patients, especially after CABG surgery.^45–47^

The findings of this network meta-analysis provide leverage in this assessment with expectable reciprocal efficacy against graft failure and risk profile. In line with the guidelines, ASA was found related to improved graft patency when compared to placebo although the confidence level for the beneficial effect was very low. As expected, the dual therapy regimens with ASA combined with ticagrelor or clopidogrel, appeared to result in even higher graft patency than other regimens especially in per-patient analysis with moderate confidence level. Ticagrelor and clopidogrel did well also as monotherapies, although there was still rather high level of uncertainty due to which more research is needed before strong recommendations on the use of ticagrelor or clopidogrel as a monotherapy against graft failure.

The graft failure rate in patients with rivaroxaban was promising alluding that rivaroxaban may perform adequately in preventing graft failure in post-CABG patients with a simultaneous need for persistent anticoagulation therapy. Interestingly, combining rivaroxaban with ASA did not seem to improve graft patency compared to rivaroxaban monotherapy advocating rivaroxaban to be used only as a monotherapy.

With regards to complications, there was a trend of increased bleeding complications and decreased MACE with each medication regimen in relation to placebo although the evidence on complication rates were mainly inconclusive. However, the observed 1-year mortality was predominantly not related to medication regimens.

### Strengths and limitations

There were several strengths in the current work. First, with regards to the previous network meta-analyses on medication regimens against graft failure in patients after CABG, this is the only work in which follow-up has been predefined to 1 year to meet the characteristics of the pathophysiological process of graft stenosis. Further, adjusting the effect estimates with potential confounders of graft patency risk enabled calculating of even more accurate effect estimates and reciprocal comparison of medication regimens. Acknowledging these strengths, the observed effect estimates were still in line with the previous network meta-analyses. The credibility of our results is affected by some limitations. First, as revealed by the risk of bias analysis, the overall risk of bias was moderate and over a half of the studies were affected by at least some concerns regarding risk of bias. In addition, with regards to some agents, the number of studies or patients was low increasing uncertainty on the effect estimate observed by wide credibility interval. There was inconsistency in the definitions and criteria for graft failure between studies. Lastly, despite the urge to control for the confounders of the association between medication regimens and graft failure, there may still be uncontrolled confounding which may have an influence on the effect estimates.

## Conclusion

As a summary, of the reviewed medication regimens, dual antiplatelet therapy combining ASA with ticagrelor or clopidogrel was found to result in the lowest rate of graft failures. These findings suggest that especially the patients with increased risk for graft failure after CABG surgery, such as in patients with acute coronary syndrome, off-pump CABG or poor graft quality, may benefit from dual antiplatelet therapy combining ASA with ticagrelor or clopidogrel.

## Supplementary Material

oeae052_Supplementary_Data

## Data Availability

The data supporting the findings of this analysis are presented in *Table 1* of this manuscript.

## Lead author biography

Dr Mikko Uimonen is a cardiothoracic surgery resident at Tampere Heart Hospital. Additionally, Dr Uimonen holds the position of Associate Professor at Tampere University. With a passion for advancing the management of coronary artery disease, Dr Uimonen is dedicated to achieving clinical excellence and conducting impactful academic research in the field of cardiothoracic surgery. His research focuses particularly on treatment selection, decision-making processes, and secondary prevention strategies, aiming to enhance patient care and outcomes through innovative approaches in cardiac surgery.
