# Supplementary material for: Preventive medication efficacy after 1-year follow-up for graft failure in coronary artery bypass surgery patients: Bayesian network meta-analysis
Source: Eur Heart J Open. 2024 Jun 27;4(4):oeae052. doi: 10.1093/ehjopen/oeae052 (PMC11227230; doi:10.1093/ehjopen/oeae052)
Supplement: oeae052_Supplementary_Data [file oeae052_supplementary_data.zip › CRD42023482354.pdf]

Preventive medication against vein graft failure after coronary artery bypass surgery – Bayesian network meta-analysis of randomized controlled trials with a minimum follow-up of a year

To enable PROSPERO to focus on COVID-19 submissions, this registration record has undergone basic automated checks for eligibility and is published exactly as submitted. PROSPERO has never provided peer review, and usual checking by the PROSPERO team does not endorse content. Therefore, automatically published records should be treated as any other PROSPERO registration. Further detail is provided here.

Citation

Ilari Kuitunen, Mikko Uimonen, Ville Ponkilainen, Rasmus Liukkonen, Matias Vaajala, Marjut Haapanen, Oskari Pakarinen, Jeremias Tarkiainen. Preventive medication against vein graft failure after coronary artery bypass surgery – Bayesian network meta-analysis of randomized controlled trials with a minimum follow-up of a year. PROSPERO 2023 CRD42023482354 Available from: https://www.crd.york.ac.uk/prospero/display_record.php?ID=CRD42023482354

Review question

What is the optimal preventive medication against vein graft failures in coronary artery bypass graft surgery patients?

Searches

We will search PubMed, Scopus, and Web of Science databases from inception to 2023. We will not use any filtering. We will not search for grey literature. We will hand search the reference lists of the included studies for potential reports. We will identify prior meta-analyses and include studies there, that we might have missed in our search process.

Types of study to be included

We will include randomized controlled trials, regardless of the blinding. The minimum follow-up needs to be at least 365 days.

Condition or domain being studied

Despite the increasing number of percutaneous coronary interventions performed worldwide, coronary artery bypass grafting (CABG) still remains a recommended treatment for patients with moderate to complex coronary artery disease. The bypassing may be performed using grafts such as internal thoracic arteries, radial artery, gastroepiploic artery or saphenous vein. Along with anastomosing left internal thoracic artery (LITA) to left anterior descending coronary artery, saphenous vein is still the most common graft for secondary anastomoses. However, anastomosing saphenous vein to coronary arteries encompasses an increased risk for graft stenosis and failure when compared to arterial grafts, and up to 30-40% of saphenous vein grafts (SVG) have been reported to fail within a year from surgery. To improve saphenous graft patency, use of antiplatelet medication, most commonly acetylsalicylic acid (ASA), is an established practice after CABG

Participants/population

Patients are required to undergo CABG surgery due to coronary artery disease. Both chronic and acute phase surgery patients will be included. CABG surgery is defined as cardiac surgery procedure in which blood flow of an occluded coronary artery has been restored by bypassing the occluded segment of the artery using a vein graft. Percutaneous coronary interventions will be excluded.

Intervention(s), exposure(s)

We will include all medical treatment regimens that are targeted against vein graft failure including agents affecting blood coagulation and thrombus formation. The minimum number of patients per treatment regimen in all included studies is set to 100 and those regimens with less than 100 patients are excluded.

Comparator(s)/control

We will use placebo as the comparator in our network meta-analysis.

Main outcome(s)

Our main outcome will be the graft failure which is defined as graft occlusion or thrombosis.

Measures of effect

Odds Ratios with 95% credible intervals.

Additional outcome(s)

Mortality, bleeding complications, major adverse cardio or cerebrovascular events

Measures of effect

Odds Ratios with 95% credible intervals.

Data extraction (selection and coding)

The following information will be extracted independently by two authors from each study: authors, funding, competing interests, inclusion and exclusion criteria, study period, country, intervention definition, control definition, outcome definitions, number of included patients, number of events and main outcome measures.

Risk of bias (quality) assessment

We will use Cochrane risk of bias 2.0 tool to addres the risk of bias in the included studies. In the risk of bias assessment, the lack of blinding was not judged as an issue as the outcome assessment was considered not to be influenced by the knowledge of the intervention

Strategy for data synthesis

We will conduct a Bayesian network meta-analysis with four Markov chains. Due to the assumed between-study heterogeneity, we select random-effects model as our approach. The posterior distributions will be estimated using Monte Carlo simulations. Simulations are performed in two phases. First, 5000 burn-in simulation iterations are performed to adapt the algorithm after which the results of these iterations are discarded. Second, 100 000 inference simulation iterations will be performed to estimate the posterior distributions. Convergence of the algorithm will be assessed by inspecting trace plots and calculating potential scale reduction factor (PSRF) values. PSFR values below 1.05 will be considered to represent sufficient convergence. Inconsistency will be assessed by conducting a node-splitting analysis. Non-significant differences ($p > 0.05$) between the direct and indirect effect estimates are interpreted as representing sufficient consistency of the network model.

After compiling the model, the crude effect estimates for each medication regimen are calculated as odds-ratios (OR) along with 95% credibility intervals (95% CrI) with placebo set as a control treatment. The rank probabilities indicating the probability for each treatment to be the most efficacious are calculated. Further, the surface under the cumulative ranking (SUCRA) scores will be calculated. Higher SUCRA score indicates higher likelihood of the medication regimen to be the most efficacious. A meta-regression analysis will be performed to adjust for the between-study differences.

Covariates to be included in the meta-regression are the mean age of patients, the proportion of female patients, mean number of grafts used, number of years from study publication and overall failure rate in each study. The 'failures per patient' and 'failures per graft' analyses will be performed separately. Sensitivity analysis on primary outcome will be performed including only low risk of bias studies.

We will use CiNeMa (Confidence in Network Meta-analyses) guidance to rate the evidence certainty.

Statistical analysis was performed using R statistical software (version 4.3.1, R Core Team (2023), R Foundation for Statistical Computing, Vienna, Austria).

This study has been conducted according to the guidelines in Cochrane handbook and reported according to the PRISMA (Preferred reporting items in systematic reviews and meta-analysis) guideline 22, 23.

Analysis of subgroups or subsets

None preplanned

Contact details for further information

Ilari Kuitunen

ilari.kuitunen@uef.fi

Organisational affiliation of the review

University of Eastern Finland

Review team members and their organisational affiliations

Dr Ilari Kuitunen. University of Eastern Finland

Dr Mikko Uimonen. Tampere Heart Hospital

Dr Ville Ponkilainen. Central Finland Hospital Nova

Mr Rasmus Liukkonen. Tampere University

Mr Matias Vaajala. Tampere University

Dr Marjut Haapanen. University of Eastern Finland

Dr Oskari Pakarinen. Tampere University

Mr Jeremias Tarkiainen. Tampere University

Type and method of review

Intervention, Network meta-analysis, Systematic review

Anticipated or actual start date

01 August 2023

Anticipated completion date

31 December 2024

Funding sources/sponsors

None to report

Conflicts of interest

Language

English

Country

Finland

Stage of review

Review Ongoing

Subject index terms status

Subject indexing assigned by CRD

Subject index terms

MeSH headings have not been applied to this record

Date of registration in PROSPERO

23 November 2023

Date of first submission

12 November 2023

Stage of review at time of this submission

**Stage**	**Started**	**Completed**
Preliminary searches	Yes	Yes
Piloting of the study selection process	Yes	Yes
Formal screening of search results against eligibility criteria	Yes	No
Data extraction	No	No
Risk of bias (quality) assessment	No	No
Data analysis	No	No

The record owner confirms that the information they have supplied for this submission is accurate and complete and they

understand that deliberate provision of inaccurate information or omission of data may be construed as scientific misconduct.

The record owner confirms that they will update the status of the review when it is completed and will add publication details in due course.

Versions

23 November 2023

23 November 2023
