# Supplementary material for: Preventive medication efficacy after 1-year follow-up for graft failure in coronary artery bypass surgery patients: Bayesian network meta-analysis
Source: Eur Heart J Open. 2024 Jun 27;4(4):oeae052. doi: 10.1093/ehjopen/oeae052 (PMC11227230; doi:10.1093/ehjopen/oeae052)
Supplement: oeae052_Supplementary_Data [file oeae052_supplementary_data.zip › Appendix1.docx]

**Appendix 1.** Complete search strategy (search date 19.2.2023)

**PUBMED**

("antithrombotic"[All Fields] OR "antithrombotics"[All Fields] OR ("antiplatelet"[All Fields] OR "antiplatelets"[All Fields]) OR ("anticoagulants"[Pharmacological Action] OR "anticoagulants"[MeSH Terms] OR "anticoagulants"[All Fields] OR "anticoagulant"[All Fields] OR "anticoagulate"[All Fields] OR "anticoagulated"[All Fields] OR "anticoagulating"[All Fields] OR "anticoagulation"[All Fields] OR "anticoagulations"[All Fields] OR "anticoagulative"[All Fields]) OR ("ticagrelor"[MeSH Terms] OR "ticagrelor"[All Fields]) OR ("clopidogrel"[MeSH Terms] OR "clopidogrel"[All Fields] OR "clopidogrel s"[All Fields]) OR ("cangrelor"[Supplementary Concept] OR "cangrelor"[All Fields]) OR ("aspirin"[MeSH Terms] OR "aspirin"[All Fields] OR ("acetylsalicylic"[All Fields] AND "acid"[All Fields]) OR "acetylsalicylic acid"[All Fields]) OR ("aspirin"[MeSH Terms] OR "aspirin"[All Fields] OR "aspirins"[All Fields] OR "aspirin s"[All Fields] OR "aspirine"[All Fields]) OR ("apixaban"[Supplementary Concept] OR "apixaban"[All Fields] OR "apixaban s"[All Fields]) OR ("rivaroxaban"[MeSH Terms] OR "rivaroxaban"[All Fields]) OR ("dabigatran"[MeSH Terms] OR "dabigatran"[All Fields] OR "dabigatran s"[All Fields]) OR ("edoxaban"[Supplementary Concept] OR "edoxaban"[All Fields]) OR ("betrixaban"[Supplementary Concept] OR "betrixaban"[All Fields]) OR ("heparin"[MeSH Terms] OR "heparin"[All Fields] OR "heparine"[All Fields] OR "heparins"[All Fields] OR "heparin s"[All Fields] OR "heparinate"[All Fields] OR "heparinated"[All Fields] OR "heparines"[All Fields] OR "heparinic"[All Fields] OR "heparinisation"[All Fields] OR "heparinised"[All Fields] OR "heparinization"[All Fields] OR "heparinize"[All Fields] OR "heparinized"[All Fields] OR "heparinizing"[All Fields]) OR ("fondaparinux"[MeSH Terms] OR "fondaparinux"[All Fields]) OR ("idraparinux"[Supplementary Concept] OR "idraparinux"[All Fields]) OR ("idrabiotaparinux"[Supplementary Concept] OR "idrabiotaparinux"[All Fields]) OR ("warfarin"[MeSH Terms] OR "warfarin"[All Fields] OR "warfarin s"[All Fields] OR "warfarinization"[All Fields] OR "warfarinized"[All Fields] OR "warfarins"[All Fields]) OR "vitamin K antagonist"[All Fields]) AND ("coronary artery bypass"[All Fields] OR "cabg"[All Fields] OR "coronary artery surgery"[All Fields] OR "coronary bypass surgery"[All Fields])

**SCOPUS:**

TITLE-ABS-KEY ( ( antithrombotic OR antiplatelet OR anticoagulant OR ticagrelor OR clopidogrel OR cangrelor OR acetylsalicylic AND acid OR aspirin OR apixaban OR rivaroxaban OR dabigatran OR edoxaban OR betrixaban OR heparin OR fondaparinux OR idraparinux OR idrabiotaparinux OR warfarin OR "vitamin K antagonist" ) AND ( "coronary artery bypass" OR "cabg" OR "coronary artery surgery" OR "coronary bypass surgery" ) ) AND ( LIMIT-TO ( DOCTYPE , "ar" ) ) AND ( LIMIT-TO ( LANGUAGE , "English" ) )

**WEB OF SCIENCE:**

antithrombotic OR antiplatelet OR anticoagulant OR ticagrelor OR clopidogrel OR cangrelor OR acetylsalicylic acid OR aspirin OR apixaban OR rivaroxaban OR dabigatran OR edoxaban OR betrixaban OR heparin OR fondaparinux OR idraparinux OR idrabiotaparinux OR warfarin OR "vitamin K antagonist") AND ("coronary artery bypass" OR "cabg" OR "coronary artery surgery" OR "coronary bypass surgery") (All Fields) and Article (Document Types) and English (Languages)
