# Supplementary material for: Preventive medication efficacy after 1-year follow-up for graft failure in coronary artery bypass surgery patients: Bayesian network meta-analysis
Source: Eur Heart J Open. 2024 Jun 27;4(4):oeae052. doi: 10.1093/ehjopen/oeae052 (PMC11227230; doi:10.1093/ehjopen/oeae052)
Supplement: oeae052_Supplementary_Data [file oeae052_supplementary_data.zip › Appendix2.docx]

**Appendix 2.** Confidence rating of effect estimates in per patient analysis for each medication regimen with placebo set as a reference according to CINeMA.

| Comparison  (Placebo set as a reference) | Number of studies | Within-study bias | Reporting bias | Indirectness | Imprecision | Heterogeneity | Incoherence | Confidence rating |
| --- | --- | --- | --- | --- | --- | --- | --- | --- |
| ASA | 3 | Major concerns | Low risk | No concerns | Major concerns | Major concerns | No concerns | **Very low** |
| Dipyridamole + ASA | 4 | Major concerns | Low risk | No concerns | No concerns | Some concerns | No concerns | **Low** |
| Acenocoumaron | 0 | Some concerns | Low risk | No concerns | Major concerns | Major  concerns | No concerns | **Very low** |
| Clopidogrel | 0 | Some concerns | Low risk | No concerns | Major concerns | Major  concerns | No concerns | **Very low** |
| Clopidogrel + ASA | 0 | Some concerns | Low risk | No concerns | No concerns | Some concerns | No concerns | **Moderate** |
| Indobufen | 0 | Some concerns | Low risk | No concerns | Major concerns | Major  concerns | No concerns | **Very low** |
| Rivaroxaban | 0 | Some concerns | Low risk | No concerns | Major concerns | Major concerns | No concerns | **Very low** |
| Rivaroxaban + ASA | 0 | Some concerns | Low risk | No concerns | Major concerns | Major concerns | No concerns | **Very low** |
| Ticagrelor | 0 | Some concerns | Low risk | No concerns | No concerns | Major concerns | No concerns | **Low** |
| Ticagrelor + ASA | 0 | Some concerns | Low risk | No concerns | No concerns | No  concerns | No concerns | **Moderate** |
